# The Diseases of Women; Their Pathology, Diagnosis and Treatment, Including the Diseases of Pregnancy

**Published:** 1883-02

**Authors:** 


**The Diseases of Women: their Pathology, Diagnosis and Treatment, Including the Diseases of Pregnancy.** By GRADY HEWITT, M. D., F. R. C. P., Lond. Fourth American from the last revised and enlarged London edition. With one hundred and thirty-two illustrations. Philadelphia: F. Blakiston, Son & Co., 1012 Walnut street. 1882. Two parts in one volume. Price, $1.50.

This is the cheap edition, in paper covers, of Dr. Hewitt's valuable work on diseases of women. We have reviewed the work in a previous number of the JOURNAL, and the favorable opinion there expressed need not be repeated here. The wide reputation of the accomplished author is a sufficient guaranty that this product of his pen will constitute an important addition to the literature of the subject or subjects on which he writes. We are yet inclined to the opinion, however, that so valuable a work should be in better binding.

